# Driving Behavior during Takeover Request of Autonomous Vehicle: Effect of Driver Postures

**DOI:** 10.3390/bs12110417

**Published:** 2022-10-28

**Authors:** Koki Muto, Shoko Oikawa, Yasuhiro Matsui, Toshiya Hirose

**Affiliations:** 1 Human Machine System Laboratory, Shibaura Institute of Technology, 3-7-5 Toyosu, Koto-ku, Tokyo 135-8548, Japan; 2 Department of Engineering Science and Mechanics, Shibaura Institute of Technology, 3-7-5 Toyosu, Koto-ku, Tokyo 135-8548, Japan

**Keywords:** driving behavior, driver posture, vehicle control, autonomous vehicle, driver monitoring

## Abstract

We investigated the effect of driver posture on driving control following a takeover request (TOR) from autonomous to manual driving in level 3 autonomous vehicles. When providing a TOR, driving behaviors need to be investigated to develop driver monitoring systems, and it is important to clarify the effect of driver postures. Experiments were conducted using driver postures that are likely to be adopted in autonomous driving. Driver postures were set based on combinations of two types of upper-body posture and three types of foot posture. The driver’s upper body and head were set to either a forward or sideways orientation. For each of these there were three types of foot posture: both feet on the floor, crossed legs, and cross-legged sitting. Following a TOR, we compared the braking and steering maneuvers of subjects driving normally and examined the effects of posture on driver reaction time. The results show that both the upper-body and foot postures of the driver affect the steering and braking reaction time. The driver monitoring system should be able to detect posture and activate a TOR warning, and detection times up to 2 and 1.3 times faster than those for normal postures should be considered for different upper-body and foot postures, respectively.

## 1. Introduction

There has been much recent research and development in the field of automated driving technology. Its practical application is expected to reduce the number of traffic accidents caused by human carelessness. For example, there were 4863 traffic accidents on highways in Japan in 2021, with 136 fatalities; of these accidents, 43% occurred owing to inattention to the road ahead, 21% owing to inattention to motion, and 14% owing to failure to see the other vehicle due to insufficient safety checks [1]. In this context, it is expected that automated driving will not only reduce the number of accidents caused through driver carelessness but also reduce the number of road deaths associated with these accidents.

The Society of Automotive Engineers (SAE) International defines six levels of autonomous driving systems, of which level 3 involves a change of control from the system to the driver under certain conditions [2]. In level 3 automated driving, the system makes a takeover request (TOR) which requires the driver to transition to manual operation when automatic driving becomes difficult because of functional limitations of the system or when the system is operating outside its design domain. The driver must be in a driving position to perform appropriate driving operations promptly when the TOR is made. The National Highway Traffic Safety Administration (NHTSA)’s guidelines for automated vehicles also state that for level 3 automated driving, the driver must be able to take control of the vehicle when notified by the vehicle [3]. Therefore, research is being conducted on the method and timing of TORs [4,5,6]. Additionally, there are different studies into ways of monitoring the driver’s condition using a driver monitoring system [7,8,9].

According to Japan’s Road Traffic Law Amendment Act, drivers may use cell phones during level 3 automated driving. However, they are required to be able to immediately perform appropriate operations when there is a transition from automated to manual driving [10]. Tasks unrelated to driving operations can affect driving behavior [11,12]. However, there are no regulations covering specific driver postures or the systems used to detect them, either in Japan’s Road Traffic Law Amendment Act or in Automated Lane Keeping Systems Regulation No. 157 of the United Nations Economic Commission for Europe (UNECE) World Forum for Harmonization of Vehicle Regulations (WP. 29) [13].

There have been tests of driver reaction times in different road conditions and in different situations, and studies on the effect of driver eye movements on reaction time [14,15]. There are also studies on driver posture [16,17]. However, few studies have investigated the relationship between driver posture and driver reaction time.

Suzuki et al. [18] studied the effects of four factors on transition time: the backrest angle, seat position, foot position, and arm position. The results showed that the driver’s foot position had the biggest effect on the braking reaction time. It was also found that the driver’s chin being placed on their hand had the biggest effect on the steering reaction time. Therefore, the relationship between driver posture and response to a TOR needs to be clarified.

In this study, we focused on driving posture and conducted experiments using a driving simulator for a level 3 autonomous vehicle. The purpose of this study was to investigate the effect of driver posture before a TOR on driver reaction after the TOR. The driver’s reaction included braking to avoid a collision with obstacles after the driving transition, and steering wheel operation to maintain the vehicle in its lane. A total of six different postures were set based on combinations of two types of upper-body posture and three types of foot posture.

## 2. Experimental Methodology

We investigated the relationship between the posture and the reaction of the driver while avoiding a collision with one or more obstacles after a TOR. The experiment assumed a situation in which a TOR happens because of the sudden appearance of an obstacle while driving in automated mode. We investigated the driver’s reaction time until the driver grasps the steering wheel after switching to manual from automated driving, as well as the braking reaction time to avoid a collision.

### 2.1. Participants

The experiment was conducted with 20 males (average age: 22), each of whom possessed a current valid driver’s license. Prior to the experiments, the purpose and procedure were explained to the participants, and their consent was confirmed. The function of the level 3 automated driving system used in this study was explained to the participants. When the system transitioned to manual operation, the participant was required to drive manually following an acoustic warning. We instructed the participants that they did not need to operate the vehicle to start, and to maintain the specified posture until the transition to manual operation was announced by an acoustic warning. Once the TOR was applied, the participants needed to operate the brakes to avoid collision with obstacles, and to steer to keep the vehicle in its lane. Participants were paid for their participation. Moreover, the experimental procedure was approved by the ethics committee of the Shibaura Institute of Technology with approval numbers of #19-014 (2019) and #20-014 (2020).

### 2.2. Driving Simulator

**Figure 1:**
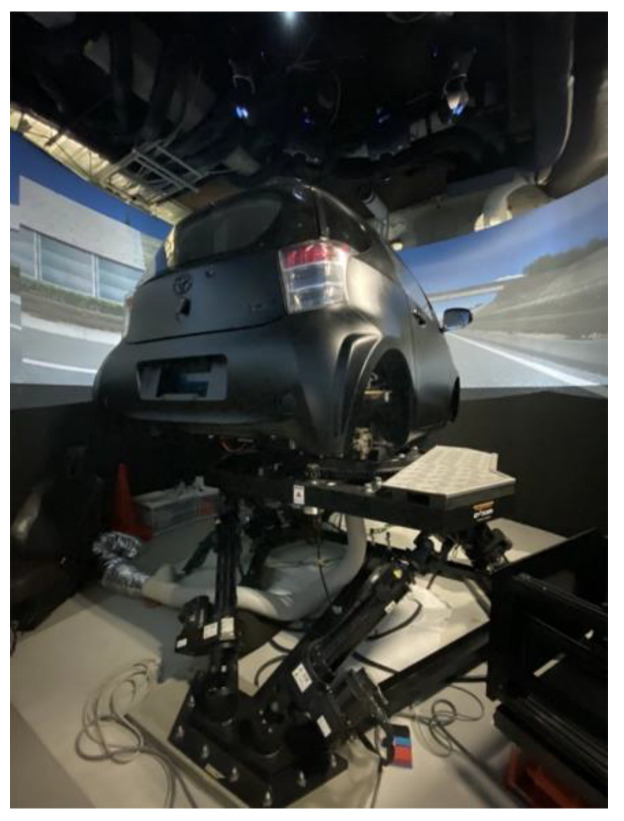
Driving simulator.

**Figure 2:**
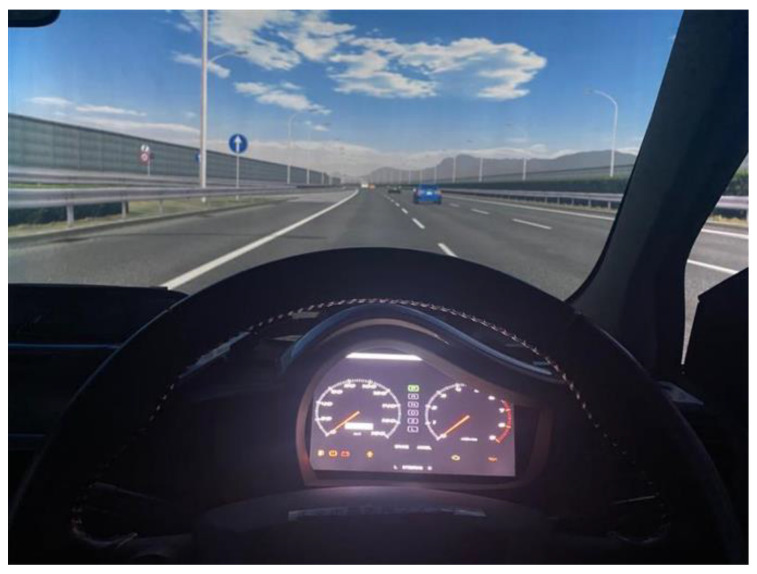
View from the driver’s seat.

A driving simulator (DS) installed at the Toyosu campus of the Shibaura Institute of Technology was used for the experiment. The DS reproduces actual motor vehicle movements via a six-axis motion device. The surrounding environment was projected onto a 360° cylindrical screen. The rear of the vehicle could be seen using the side and rear-view mirrors installed in the cabin. In addition, an image of the rear view was projected onto the screen. A six-axis motion device equipped with electric actuators was used to simulate the vehicle motion; specifically, the DS could simulate the rolling motion caused by steering wheel action, and the pitching motion caused by the braking of the vehicle. Moreover, the yawing motion of the vehicle could be simulated using a turntable able to rotate 180° to the left and right, on top of the six-axis motion device, to match the driver’s steering wheel action. The DS braking system consists of a brake pedal, brake booster, rotor, and caliper. Pressure is applied to the brake booster to simulate the sensation of a pedal during braking. The DS steering wheel was equipped with a high-speed brushless DC servomotor, manufactured by Moog, to impart the appropriate steering reaction force during operation, the reaction force being simulated by a real-time computer. This enabled the driver to feel the steering reaction force, calculated according to the vehicle motion. The specifications of the six-axis motion device and the turntable of the DS are shown in Table 1. Figure 1 shows an image of the exterior of the simulator, and Figure 2 shows an image of the front screen taken from the driver’s seat.

### 2.3. Driver Posture

We conducted the experiment using driver postures that are likely to be adopted during autonomous driving. The driving posture parameters consisted of upper-body posture (seated and forward-facing and seated and sideways-facing) and foot posture (both feet on the floor (BFF), crossed legs (CL), and cross-legged sitting (S)). The six different combinations of postures were set as shown in Table 2.

We set the forward and sideways-facing postures as the state of the upper body. The forward posture was defined as looking as far forward as possible, with hands resting on the thighs. The sideways posture was defined as facing the passenger seat, as if talking to a passenger. This was based on a questionnaire study on automated driving conducted in Germany [19]. To the question “what do you think you will do under highly automated driving?”, the most popular response was “talk with passengers” (90.3%). In this experiment, the driver’s seat was on the right-hand side of the vehicle. When the direction of travel was forward, the driver faced left in a sideways-facing posture. The participants were not actually talking but were asked to look toward the passenger seat to simulate their posture during a conversation and place both hands on the seat belt receptacle notch of the passenger seat.

Passengers are expected to assume a relaxed posture during automatic operation. Kobayashi and Muranaka [20] showed that in a restaurant, 24% of customers who were seated at a table had their legs crossed. In addition, zazen (Zen) is a body posture similar to forward-S (defined above). In zazen, people feel relaxed [21]. While the S posture is a relaxed foot posture, it is also the most difficult from which to change and return to the normal foot posture. Therefore, the experimental conditions for this study were (1) BFF, (2) CL, and (3) S. In the BFF posture, both feet were placed on the floor together. Since the driver operates the accelerator and brake pedals with their right foot, in the CL posture, the right foot was placed on the driver’s seat and folded under the left leg. In the S posture, both feet were placed on the seat surface, with the right foot placed near the driver’s body and the left foot crossed over the right.

### 2.4. Scenarios

**Figure 3:**
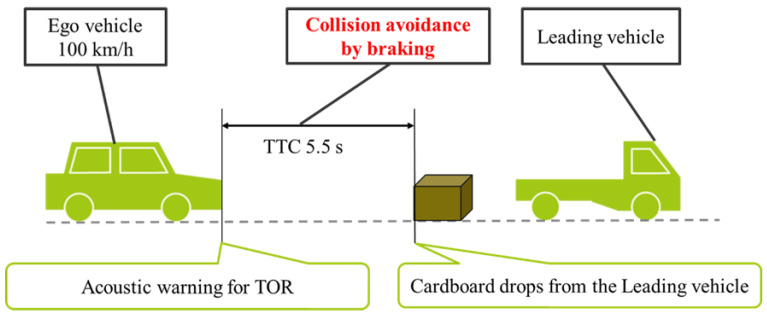
Takeover request (TOR) acoustic warning scenario.

In this study, experiments were conducted on a course that simulated a three-lane road (lane width 3.8 m) in the same direction as the Tomei Expressway in Japan. In the experiment, we simulated a situation in which the ego vehicle was traveling under automated control on the highway when a cardboard box (an obstacle) suddenly fell from a truck ahead. The instant the automated driving system detected the falling obstacle, a warning was given to inform the driver about the impending transition to manual driving. In the experiment, the obstacle fell while both vehicles were traveling at 100 km/h. Obstacles were dropped at random locations to reduce the learning effect on the experiment participants. The timing of the acoustic warning for switching to manual driving was set to when the time-to-collision (TTC) was 5.5 s. The TTC is the time taken for the ego vehicle to reach the point of a potential collision with the obstacle under constant speed, as shown in Figure 3. This value was set according to the value that received the highest subjective evaluation in a previous study investigating the optimal transition time from automatic to manual operation [22]. The acoustic warning was a single tone that was played twice in succession. The loudness of the warning sound was 88.3 dB, while the normal driving noise level was 67.6 dB. Before starting the experiment, we explained to the participants that when the acoustic warning was sounded, the transition to manual driving would occur.

In this experiment, the collision-avoidance driving behavior included a braking maneuver and grasping of the steering wheel to maintain lane control. The reason for limiting the driving behavior to braking operations is that this experiment assumes the highest risk situation. A braking maneuver takes longer than a steering maneuver to avoid collisions with obstacles when a vehicle is traveling at high speeds [23]. In addition, the driver may not be able to steer the vehicle if the next lane is occupied by other vehicles. Therefore, we targeted braking maneuvers as maneuvers for collision avoidance with obstacles. Participants were required to operate the brake pedal until the vehicle had come to a complete stop after the acoustic warning was sounded. The experiment started with automated driving, and then the participants were required to adopt a specified posture and maintain it until the TOR. To minimize the influence of the same posture on the driving behavior over long periods of time, the scenarios were set so that the duration of each experiment was approximately 3 min. A total of six experiments were conducted for each participant. Before the start of the experiment, the participants underwent one practice run to familiarize themselves with driving operations of the simulator.

### 2.5. Evaluation Items

To investigate the effect of driver posture on driving behavior during automated driving, the evaluation items were the braking reaction time, steering reaction time, and obstacle distance. The braking reaction time was defined as the time taken by the driver to place their foot on the brake after the transition to manual driving. The time taken to place their foot on the brake was chosen to measure the time taken to prepare for braking based on the acoustic warning during the transition. The reaction time was measured as the time at which the numerical value of the rotation angle meter attached to the brake pedal of the DS started to change.

The steering reaction time was defined as the time from the transition to manual driving until the driver regained control of the steering wheel. The steering reaction time was analyzed by video, instead of by change in the steering angle because there could have been cases in which the steering wheel was not operated immediately after being grasped by the driver. The ultra-sensitive monochrome camera (the WAT-910HX MBD, manufactured by Watec Co., Ltd., Yamagata, Japan), which measured the steering operation, had dimensions of 23 mm × 23 mm × 23.8 mm. The minimum subject illuminance of the video camera was 9 × 10^−5^ lx, and the f-number of the lens was 2.0, which was suitable for shooting in the dark. Consequently, it was possible to observe the driver without affecting the driving operation. The data from the video camera were stored in real-time on a recorder (the WJ-NX400, manufactured by Panasonic Connect Co., Ltd., Tokyo, Japan), and the reaction time of the driver was analyzed based on this data.

In this study, the distance between the obstacle and the vehicle was measured to evaluate the driver’s braking operation for collision avoidance with the obstacle. This distance was defined as the distance from the front of the vehicle to the rear of the obstacle, calculated from the driving data recorded in the DS.

The mean values for braking reaction time, steering reaction time, and obstacle distance were calculated from the data of the 20 participants. A significant difference test (Wilcoxon’s signed rank test) was conducted for each of the six driving postures.

## 3. Results

### 3.1. Braking Reaction Time

**Figure 4:**
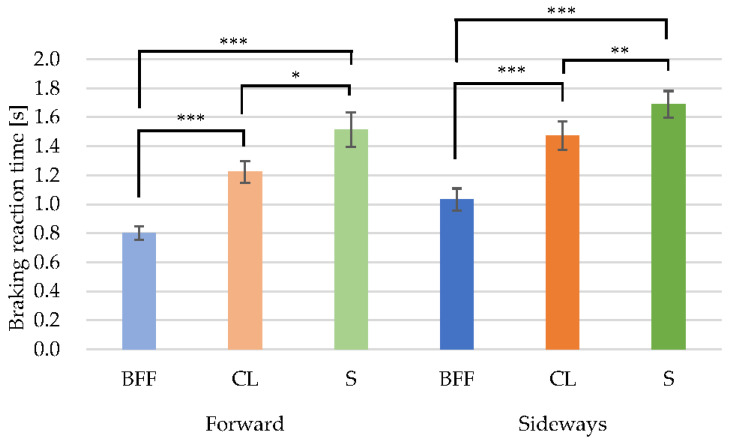
Foot posture comparisons of braking reaction time. * *p* < 0.05/** *p* < 0.01/*** *p* < 0.001.

Comparisons were made using significance difference tests in each posture. Table 3 shows the results, comparing the forward-facing and sideways-facing postures of the upper body. Table 4 is a quartile table of brake reaction times. The sideways posture was significantly slower than the forward posture for all foot postures. When compared with each other for the same foot posture, the forward-S posture was significantly slower than the forward-CL posture (*p* = 0.017), and the forward-CL posture was significantly slower than the forward-BFF posture (*p* < 0.001) (Figure 4).

The sideways-S posture was significantly slower than the sideways-CL posture (*p* < 0.001), the sideways-S posture was significantly slower than the sideways-BFF posture (*p* < 0.001), and the sideways-CL posture was significantly slower than the sideways-BFF posture (*p* < 0.001) (Figure 4).

Compared with the forward-BFF posture, the breaking reaction times of the forward-CL and -S postures were about 1.5 and 1.9 times longer, respectively. For the sideways posture, the breaking reaction times of the CL and S postures were 1.4 and 1.6 times longer than that of the BFF posture, respectively. In the sideways postures, it took 1.1–1.3 times longer to respond than in the forward postures.

### 3.2. Steering Reaction Time

**Figure 5:**
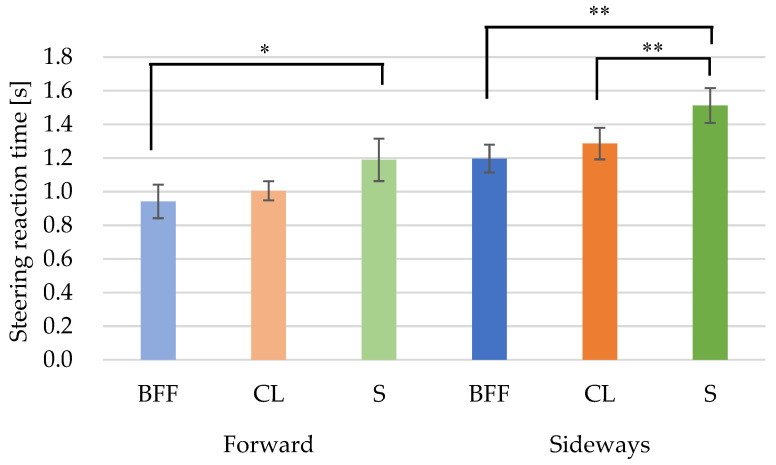
Foot posture comparisons of steering reaction time. * *p* < 0.05/** *p* < 0.01.

Table 5 shows the results of comparing the forward-facing and sideways-facing postures of the upper body. Table 6 is a quartile table of brake reaction times. In comparing the two different upper-body postures, the sideways postures were found to be significantly slower than the forward postures for all foot postures. When compared with each other for the same foot postures, the forward-S posture was slower than the forward-CL posture, but not significantly so, and it was significantly slower than the forward-BFF posture (*p* = 0.015) (Figure 5).

The sideways-S posture was significantly slower than the sideways-CL posture (*p* = 0.004), the sideways-S posture was significantly slower than the sideways-BFF posture (*p* = 0.002), and the sideways-CL posture was slower than the sideways-BFF posture, but not significantly so (Figure 5).

For both the forward and sideways postures, drivers in the CL and S postures took about 1.1 and 1.3 times longer to respond, respectively, than when in the BFF posture. In addition, the steering reaction time of the sideways postures was about 1.3 times longer than those of the forward postures.

### 3.3. Comparison of Braking Reaction Time and Steering Reaction Time

The braking versus steering reaction times for the forward-BFF (0.80 s versus 0.94 s) and sideways-BFF (1.03 s versus 1.2 s) postures suggest that the mean breaking reaction time was faster than the mean steering reaction time. Conversely, for the forward-CL (1.22 s versus 1.01 s), sideways-CL (1.47 s versus 1.29 s), forward-S (1.52 s versus 1.19 s), and sideways-S (1.69 s versus 1.51 s) postures, the mean steering reaction time was faster than the braking reaction time.

### 3.4. Distance from the Ego Vehicle to the Obstacle

Table 7 shows comparisons of the forward and sideways postures. The distance for the sideways posture was significantly shorter than for the forward posture for all foot postures.

**Figure 6:**
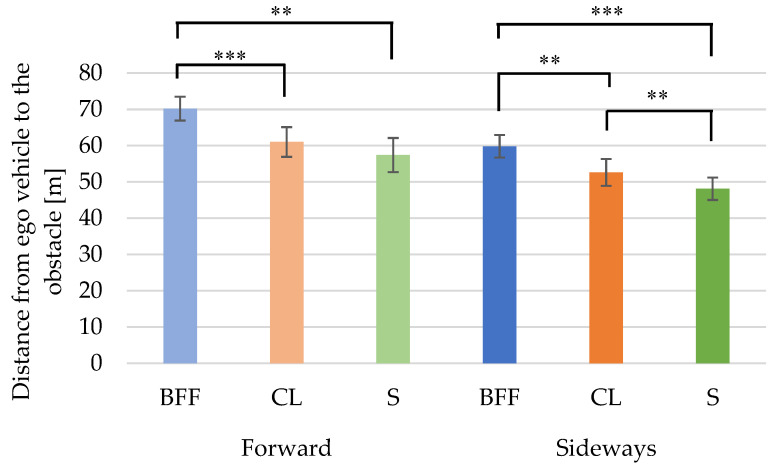
Leg posture comparisons of distance from the ego vehicle to the obstacle. ** *p* < 0.01/*** *p* < 0.001.

The forward-S posture was significantly shorter than the forward-BFF posture (*p* = 0.009). The forward-CL posture was significantly shorter than the forward-BFF posture (*p* < 0.001) (Figure 6).

The sideways-S posture was significantly shorter than the sideways-CL posture (*p* = 0.009), the sideways-S posture was significantly shorter than the sideways-BFF posture (*p* < 0.001), and the sideways-CL posture was significantly shorter than the sideways-BFF posture (*p* = 0.007) (Figure 6).

## 4. Discussion

### 4.1. Reaction Time

Comparing the forward-BFF (0.80 s) and sideways-BFF (1.03 s) postures, the forward-CL (1.22 s) and sideways-CL (1.47 s) postures, and the forward-S (1.52 s) and sideways-S (1.51 s) postures, it was found that the braking response was slower in the sideways position. This could be because the drivers tended to be conscious of steering in the sideways position in a situation in which it was difficult to shift to the steering action itself. In fact, 10 of the 20 participants (50%) showed slower braking responses in the sideways posture compared with the forward posture for each of the foot postures. The results obtained in this study are consistent with those reported by Wu et al., which showed that 29 out of the 36 participants avoided a collision due to the sudden appearance of an obstacle by steering only when the TOR warning was sounded [15]. This suggests that the driver may prioritize steering in TOR warning situations.

In both the forward and sideways postures, the steering reaction time was slower in the S posture (forward: 1.19 s; sideways: 1.51 s) than in the CL posture (forward: 1.01 s; sideways: 1.29 s), and slower in the CL posture (forward: 1.01 s; sideways: 1.29 s) than in the BFF posture (forward: 0.94 s; sideways: 1.2 s). Comparing the forward-BFF (0.94 s), CL (1.01 s), and S (1.19 s) postures with the sideways-BFF (1.20 s), CL (1.29 s), and S (1.51 s) postures, respectively, shows that the S posture significantly delays the steering reaction time. This suggests that when the driver was in a situation in which it was difficult to shift to braking, the steering wheel grasp may also have been delayed. These results suggest that the condition of the driver’s upper body may have affected the steering as well as the braking. Similarly, the driver’s foot posture affected not only the braking, but also the steering.

In fact, the steering reaction times for the sideways-BFF and the forward-S postures were similar. Therefore, even if the upper body is forward-facing, the amount of influence on the steering reaction time is equivalent to the sideways posture (BFF) if the subject is in an S posture.

Braking reaction times were compared with the results from previously reported experiments [14]. In the compared experiments, the driver’s collision avoidance was mostly limited to braking because of the difficulty of steering, due to vehicles in the adjacent lane. The median brake reaction time for collision avoidance ranged from 0.81 s to 1.15 s, depending on the distance from the vehicle ahead (10–50 m). In this experiment, the median braking reaction time ranged from 0.79 s to 1.67 s, indicating that the effect of driving posture on the reaction time was greater than in other experimental conditions.

### 4.2. Comparison of Obstacle Distance Results and Brake Reaction Time Results

For the upper-body postures, the significant differences between the forward-BFF and sideways-BFF postures and the forward-CL and sideways-CL postures were 5% in obstacle distances and 1% in braking reaction times.

As for the obstacle distance, it was found that the distance of the forward-CL posture was shorter than that of the forward-BFF posture at the 0.1% level, and that of the forward-S posture was shorter than that of the forward-CL posture. Similarly, the sideways-CL posture distance was shorter than the sideways-BFF posture distance at the 1% level, and the sideways-S posture distance was shorter than the sideways-CL posture distance at the 1% level. Those with different levels of significant differences in obstacle distance and braking reaction time were forward-BFF vs. forward-S and sideways-BFF vs. sideways-CL. The results with a higher level of significance for brake reaction time than for obstacle distance showed that the foot posture had an effect on the obstacle distance, and less so on the braking reaction time. The drivers in the forward-S and sideways-CL postures might control the brakes more ably to avoid collisions with obstacles than those in the forward-BFF and sideways-BFF postures because of their delayed braking time.

These results suggest that the upper-body and foot postures of drivers are more likely to have an effect on the braking reaction time than the obstacle distance.

### 4.3. Driving Monitoring System

According to the Automated Lane Keeping System regulation UN-R157 [13], an automated driving system should include a driver availability recognition system, which can detect whether the driver is ready to take over the driving operation. Therefore, it is important to investigate the driver’s posture during autonomous driving to ensure that the driver can properly take over the driving operation.

Compared with the forward-BFF posture, in which the driver’s right foot can immediately apply the brake, the braking reaction time in the sideways-CL posture, in which the driver’s body is turned sideways, were more than twice as long. In actual driving, the driver is expected to face different situations, such as talking with passengers, using a cell phone, or eating and drinking during autonomous driving. Therefore, the reaction time may be extended accordingly. Some current autonomous driving systems monitor the state of the driver’s eyes and head, but the results of this experiment suggest that it is necessary to monitor the state of the driver’s feet and body as well. The driver monitoring system should be able to detect posture and activate a TOR warning, and detection times up to 2 and 1.3 times faster should be considered for different foot (CL, S) and upper-body (sideways) postures, respectively, when compared with normal postures (BFF, forward).

The results of this experiment will contribute to the determination of the detection time for the driver’s driving-ready posture by a driver monitoring system.

### 4.4. Limitations

We were not able to investigate the driving characteristics of the elderly because only people in their 20 s participated in this experiment. It would be necessary to consider the age of participants in future experiments. The differences in driving characteristics between younger and older drivers after the transition from automated to manual driving have been investigated. Peng and Iwaki [24] found that elder drivers had higher crash rates and less stable lateral control than younger drivers when faced with a TOR.

In this experiment, participants were expected to avoid collisions only by braking, and we were not able to verify the results under conditions in which steering operation was possible. Since drivers also avoid collisions by steering in actual driving conditions, it would be necessary to investigate the effect of steering on driving characteristics under conditions wherein collision avoidance may be taught in the future.

In an actual driving environment, drivers may also operate cell phones, eat, and drink. There is a possibility that they would adopt postures other than those targeted in this study. It would also be necessary to examine the effects of such behaviors on driving characteristics.

This experiment was conducted using the DS method, where the behavioral patterns in risk recognition and TOR warning situations are constant. Therefore, it would be necessary to consider experiments better adapted to real environments.

In this experiment, we did not vary the TTC with obstacles, which was kept at 5.5 s. If the TTC were changed, it is possible that the resulting operating characteristics after the transition to manual operation may differ from the results of this experiment.

The results obtained from these experiments can be used as a reference in the design of driver monitoring systems. In future studies, it will be necessary to investigate the design requirements for driver monitoring systems.

## 5. Conclusions

Driving behavior should be studied to develop driver monitoring systems for level 3 autonomous vehicles and safely provide TORs.

Therefore, the purpose of this study was to investigate the effect of driver posture before the transition from automated driving to manual driving on driving behavior after the transition. Six different postures were used, consisting of combinations of two types of upper-body postures and three types of foot postures.

From the experimental results, it was found that both the upper-body and foot postures of the driver affected the steering and braking reaction time. The driver monitoring system should be able to detect posture and activate the warning for a TOR, and detection times up to 2 and 1.3 times faster when compared with normal postures should be considered for different upper-body and foot postures, respectively. Additionally, the sideways-S posture, which had the greatest impact on control in this position, should be actively discouraged during level 3 automated driving. Since posture also affects the driver’s behavior after a TOR, these results are expected to contribute to the construction of a driver monitoring system able to detect postures that affect driving characteristics and able to give the driver an appropriate warning.

## Figures and Tables

**Table 1:** Specification of the driving simulator and turntable.

**Driving Simulator**		
Power supply	AC 200 V	
Driving mass		1000 [kg]
Range of movement	X	+116/−106 [mm]
	Y	±104 [mm]
	Z	+114/−104 [mm]
	YAW	±7.3 [deg]
	ROLL	±7.9 [deg]
	PITCH	+7.8/−8.3 [deg]
Maximum speed	X	260 [mm/s]
	Y	250 [mm/s]
	Z	257 [mm/s]
	YAW	17 [deg/s]
	ROLL	18 [deg/s]
	PITCH	19 [deg/s]
Maximum acceleration	X	±0.6 [G]
	Y	±0.6 [G]
	Z	±0.5 [G]
**Turntable**		
Driving moment of inertia	200 [kg m^2^]	
Range of movement	±180 [deg]	
Maximum angular velocity	±60 [deg/s]	
Weight	500 [kg]	

**Table 2:** Posture parameters.

Upper Body	Forward			Sideways		
Foot/Leg ^1^	BFF	CL	S	BFF	CL	S
Posture	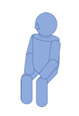	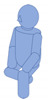	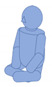	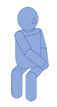	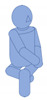	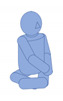

^1^ BFF: Both feet on the floor, CL: Crossed legs, S: Cross-legged sitting.

**Table 3:** Braking reaction times.

Foot Position	Braking Reaction Times [s]						Forward vs. Sideways Ratio ^(2)^	*p*-Value
	Forward			Sideways				
	Average	BFF vs. CL or S Ratio ^(1)^	SD	Average	BFF vs. CL or S Ratio ^(1)^	SD		
BFF	0.8	-	0.05	1.03	-	0.08	1.3	0.006 **
CL	1.22	1.5	0.08	1.47	1.4	0.1	1.2	0.003 **
S	1.52	1.9	0.12	1.69	1.6	0.09	1.1	0.015 *

^(1)^ $\;Ratio=\frac{Average\;reaction\;time\;of\;CL\;or\;S}{\;Average\;reaction\;time\;of\;BFF}\;$^(2)^ $\;Ratio=\frac{Average\;reaction\;time\;posturing\;sideways}{\;Average\;reaction\;time\;posturing\;forward}$ * *p* < 0.05/** *p* < 0.01.

**Table 4:** Quartile table of brake reaction times.

Posture	Braking Reaction Times [s]					
	Forward			Sideways		
	First Quartile	Median	Third Quartile	First Quartile	Median	Third Quartile
BFF	0.66	0.79	0.96	0.81	0.98	1.12
CL	1.07	1.19	1.51	1.18	1.38	1.76
S	1.13	1.52	1.80	1.41	1.67	1.90

**Table 5:** Steering reaction times.

Foot Position	Steering Reaction Times [s]						Forward vs. Sideways Ratio ^(2)^	*p*-Value
	Forward			Sideways				
	Average	BFF vs. CL or S Ratio ^(1)^	SD	Average	BFF vs. CL or S Ratio ^(1)^	SD		
BFF	0.94	-	0.1	1.2	-	0.08	1.3	0.004 **
CL	1.01	1.1	0.06	1.29	1.1	0.09	1.3	0.002 **
S	1.19	1.3	0.13	1.51	1.3	0.1	1.3	<0.001 ***

^(1)^ $\;Ratio=\frac{Average\;reaction\;time\;of\;CL\;or\;S}{\;Average\;reaction\;time\;of\;BFF}\;$^(2)^ $\;Ratio=\frac{Average\;reaction\;time\;posturing\;sideways}{\;Average\;reaction\;time\;posturing\;forward}$ ** *p* < 0.01/*** *p* < 0.001.

**Table 6:** Quartile table of steering reaction times.

Posture	Steering Reaction Times [s]					
	Forward			Sideways		
	First Quartile	Median	Third Quartile	First Quartile	Median	Third Quartile
BFF	0.63	0.85	1.11	0.97	1.07	1.32
CL	0.86	1.02	1.13	1.07	1.32	1.46
S	0.83	1.00	1.37	1.19	1.37	1.76

**Table 7:** Distance from the ego vehicle to the obstacle.

Foot Position	Forward		Sideways		*p*-Value
	Distance from the Ego Vehicle to the Obstacle [m]	SD	Distance from the Ego Vehicle to the Obstacle [m]	SD	
BFF	70.2	3.3	59.8	3.1	0.013 *
CL	61.0	4.1	52.6	3.7	0.016 *
S	57.4	4.7	48.1	3.1	0.020 *

* *p* < 0.05.

## Data Availability

The data used to support the findings of this study are available from the corresponding author upon request.
